# Delayed lead straight punch performance following resistance priming with different velocity-loss thresholds in highly trained boxers

**DOI:** 10.3389/fphys.2026.1828882

**Published:** 2026-09-03

**Authors:** Yinkai Zhang, Xiaoyu Liu, Jinxuan Bao, Xinbi Zhang, Penglin Diao, Guoding Sun, Jintao Zhang, Qingmin Fan, Ziyu Wang, Juan Del Coso

**Affiliations:** 1 Sports Coaching College, Beijing Sport University, Beijing, China; 2 China Wushu School, Beijing Sport University, Beijing, China; 3 School of Arts and Sciences, Fuyao University of Science and Technology, Fuzhou, China; 4 School of Strength and Conditioning Training, Beijing Sport University, Beijing, China; 5 School of Kinesiology and Health, Capital University of Physical Education and Sports, Beijing, China; 6 School of Sports Training, Wuhan Sports University, Wuhan, China; 7 Faculty of Health, University of Canterbury, Christchurch, New Zealand; 8 Sport Sciences Research Centre, Rey Juan Carlos University, Fuenlabrada, Spain; 9 Faculty of Health Sciences, Institute of Health and Sport Sciences, Universidad Francisco de Vitoria, Madrid, Spain

**Keywords:** autoregulation, combat sports performance, neuromuscular fatigue, resistance priming, strength-velocity relationship, upper-body power

## Abstract

**Background:**

Optimizing pre-competition priming is critical for maximizing acute performance in combat sports. Traditional percentage-based training (PBT), which uses a fixed percentage of one-repetition maximum (1RM) for a set number of repetitions, is widely used but lacks autoregulation, potentially causing suboptimal stimulus or excessive fatigue. In contrast, velocity-based training (VBT) adjusts training volume by ending sets when a prescribed velocity loss (VL) threshold is reached, providing individualized fatigue management. This study examined delayed punch-performance responses following traditional PBT and VBT performed with VL thresholds of 10%, 20%, or 30%, with each active condition evaluated relative to a low-intensity active control condition.

**Methods:**

Eighteen highly trained male boxers completed five priming conditions in a randomized crossover design, each separated by approximately 5 days: a low-intensity active control condition (CON; bench press at <30% 1RM for 3 sets of 10 repetitions with 3 min rest between sets), percentage-based training (PBT; 3 sets of 5 repetitions at 85% 1RM), and velocity-based training conditions performed at 85% 1RM with sets terminated at 10%, 20%, or 30% velocity loss. In the VBT conditions, barbell velocity was monitored on each repetition, and sets were stopped when mean concentric velocity dropped by the target percentage. Lead straight punch performance was assessed using relative peak punch force (RPF; N/kg), peak punch velocity (PPV; m/s), and an estimated relative punch-power index (RPP; W/kg) at baseline and 6 h, 24 h, and 48 h after priming.

**Results:**

The condition × time interactions were not significant for RPF, PPV, or RPP. At baseline, no active condition differed significantly from CON. At 6 h, RPP was significantly higher than CON following PBT, VL10%, and VL20%, whereas PPV was higher than CON following VL10% and VL20%; RPF did not differ significantly from CON. At 24 h, RPP was higher than CON following all active conditions; PPV was higher than CON following PBT, VL10%, and VL20%; and RPF was higher than CON following VL10%, VL20%, and VL30%. At 48 h, no active condition differed significantly from CON.

**Conclusion:**

At specific post-intervention time points, VL10% and VL20% showed higher PPV and/or RPP values than the low-intensity active control condition at 6 h and 24 h, whereas VL30% showed higher RPF and RPP values than CON at 24 h. Because the condition × time interactions were not significant and change-from-baseline contrasts were not performed, these findings represent time-specific differences from CON rather than confirmed differences in change from baseline or temporal response. The comparisons also do not establish direct differences among the active protocols, and the unequal training doses across VL conditions preclude identification of an optimal VL threshold.

## 1 Introduction

Optimizing acute physical function during the pre-competition phase is essential for maximizing sport-specific performance in athletes (Mason et al., 2020). Emerging evidence indicates that preparatory strategies implemented before or around competition may acutely influence short-term sport-specific performance (Blazevich and Babault, 2019). As a high-intensity combat sport, boxing demands rapid muscle activation and coordinated force production to deliver effective strikes, making striking power a critical performance attribute (Kirk et al., 2021). In particular, the lead straight punch, a fundamental technique used for offensive, defensive, and tactical actions, depends on the effective transmission of force through the kinetic chain involving the lower limbs, trunk, and upper limbs (Piorkowski et al., 2011). Accordingly, training approaches that integrate both strength and movement velocity may enhance kinetic-chain coordination, such that punching power reflects the cumulative contribution of the entire body rather than isolated upper-limb strength (Dinu and Louis, 2020; Cui et al., 2024).

To evaluate the effectiveness of pre-competition priming strategies on boxing performance, punching performance can be quantified using measures of impact force, punch velocity, and an estimated force–velocity product. Accordingly, relative peak punch force (RPF; N/kg), peak punch velocity (PPV; m/s), and an estimated relative punch-power index (RPP; W/kg) were used as practical markers of boxing-specific explosive performance (Loturco et al., 2016; Beattie and Ruddock, 2022). Body-mass normalization of RPF and RPP improves comparability across athletes from different weight categories, whereas PPV is expressed in absolute units (m/s) (Dunn et al., 2022). These metrics capture key neuromuscular qualities targeted by priming interventions, as they are closely related to the neuromuscular system’s ability to produce high force under high-velocity movement conditions (Loturco et al., 2016; Liu et al., 2022). Upper-body pushing strength and power are associated with punch-impact metrics in trained boxers, supporting the bench press as a practical exercise for priming and training (Dunn et al., 2022). Consequently, appropriately designed strength training may positively influence neuromuscular characteristics underlying force and velocity production, which are relevant to lead straight punch performance (Pareja-Blanco et al., 2017; Mosler et al., 2024). Therefore, optimizing striking performance through the effective management of training load and neuromuscular fatigue has become a central focus of contemporary boxing training research (Uthoff et al., 2023).

Delayed Performance Enhancement (DPE) refers to improvements in athletic performance that occur approximately 6–48 h after a high-intensity, low-volume neuromuscular stimulus (e.g., strength, sprint, or high-intensity endurance activities), and is conceptually distinct from Post-Activation Performance Enhancement (PAPE), which reflects transient performance increases observed within minutes following a conditioning activity (Blazevich and Babault, 2019). This is why PAPE is generally implemented immediately (i.e., minutes) prior to performance, whereas DPE is incorporated into pre-competition training (i.e., hours) to enhance performance following a period of recovery (Boullosa, 2021). DPE reflects a complex neuromuscular adaptation process within the training-recovery cycle (Panteli et al., 2024). Unlike immediate performance enhancements observed during or shortly after warm-up activities, DPE manifests after a period of recovery and reflects the dynamic interplay between training-induced neuromuscular stimulation and the resolution of the fatigue induced by the training. Potential mechanisms for DPE include the combined effects of several factors, such as enhanced central drive, improved motor unit recruitment efficiency, enhanced excitation-contraction coupling, and muscle glycogen supercompensation. High-intensity stimulation has been hypothesized to transiently alter neural excitability and motor-unit recruitment, although the physiological mechanisms underlying DPE remain uncertain. Additionally, adequate recovery and nutritional intake may facilitate the restoration of intramuscular energy substrates, potentially supporting subsequent high-intensity performance (González-García et al., 2021). The DPE time course and mechanisms contrast with PAPE, which stems from transient mechanisms (Seitz and Haff, 2016; Blazevich and Babault, 2019). Given these distinct temporal and mechanistic characteristics of DPE vs PAPE, recent research has increasingly examined the magnitude and time course of DPE across different resistance/exercise training protocols.

Traditional strategies for inducing DPE rely primarily on percentage-based training (PBT), where athletes perform several repetitions of a given resistance exercise at approximately 85–90% of the one-repetition maximum (1RM) 1–2 days before competition (Nishioka and Okada, 2023; Niu et al., 2025). However, PBT is unable to accommodate daily fluctuations in 1RM before competition due to factors like tapering, fatigue or sleep, risking either excessive fatigue or insufficient stimulus (González-Badillo and Sánchez-Medina, 2010; Weakley et al., 2021). Furthermore, it cannot provide real-time feedback on fatigue accumulation, which makes it difficult to promptly modify training volume based on actual power output. In recent years, velocity-based training (VBT) has emerged as a promising priming alternative for inducing DPE, as it dynamically adjusts load and volume based on real-time movement velocity fluctuations (González-Badillo and Sánchez-Medina, 2010; Sánchez-Medina and González-Badillo, 2011). In VBT, the velocity loss (VL) threshold determines training dose, with lower thresholds (≤20%) preserving power output and higher thresholds (≥30%) inducing greater fatigue (Hernández-Belmonte and Pallarés, 2022; Jukic et al., 2023). Accordingly, VBT may offer a more precise and individualized strategy for optimizing the stimulus–fatigue balance needed for inducing DPE during pre-competition priming (Liao et al., 2021).

Recent evidence further indicates that movement intent and contraction velocity are important determinants of power-oriented adaptations. In elite athletes performing volume-load-matched resistance training, maximal-intended-velocity training produced greater improvements in mean propulsive velocity and power than controlled-velocity training, despite similar improvements in maximal strength. Subsequent evidence also showed that ballistic training performed with maximal movement intent improved maximal propulsive power, rate of force development, and impulse, with improvements in maximal propulsive power being positively associated with changes in rate of force development. Collectively, these findings support the use of velocity monitoring and VL thresholds to preserve high-velocity contractions when rapid force and power production are the primary training objectives (Lecce et al., 2025a; Lecce et al., 2025b).

Currently, only a few studies have examined the immediate effect of different VL thresholds on PAPE in boxing, showing a transient increase in punch power 8–12 minutes post-exercise (Cui et al., 2024). However, the DPE effects of VL strategies on punching performance remain unexplored, including whether such pre-stimulation can enhance performance overnight and how fatigue modulation influences this process. Additionally, current research has predominantly examined DPE using resistance priming protocols and assessed outcomes through lower-limb performance measures (e.g., countermovement jump height or sprint speed), consistently demonstrating enhanced explosive performance within 6–48 h post-training (Harrison et al., 2019; González-García et al., 2021; Panteli et al., 2024). However, the effects of DPE on upper-limb, sport-specific actions, particularly boxing strikes, remain insufficiently investigated. Boxing performance depends on rapid, coordinated force transmission across the entire kinetic chain rather than isolated vertical force production typical of lower-limb tasks. Given these biomechanical and neuromuscular differences, it remains unclear whether findings from lower-limb DPE research translate to punching performance in boxers.

Based on this rationale, the present study aimed to examine the DPE effects of distinct resistance priming protocols—comprising three VBT conditions with different velocity-loss thresholds (VL10%, VL20%, and VL30%) and one traditional PBT condition—on lead straight punch performance in highly trained boxers. It was hypothesized that VBT performed with low-to-moderate velocity-loss thresholds (VL10% and VL20%) would demonstrate favorable delayed lead straight punch performance responses relative to the low-intensity active control condition, particularly within the 6–24 h post-training window.

## 2 Methods

### 2.1 Experimental design

**Figure 1:**
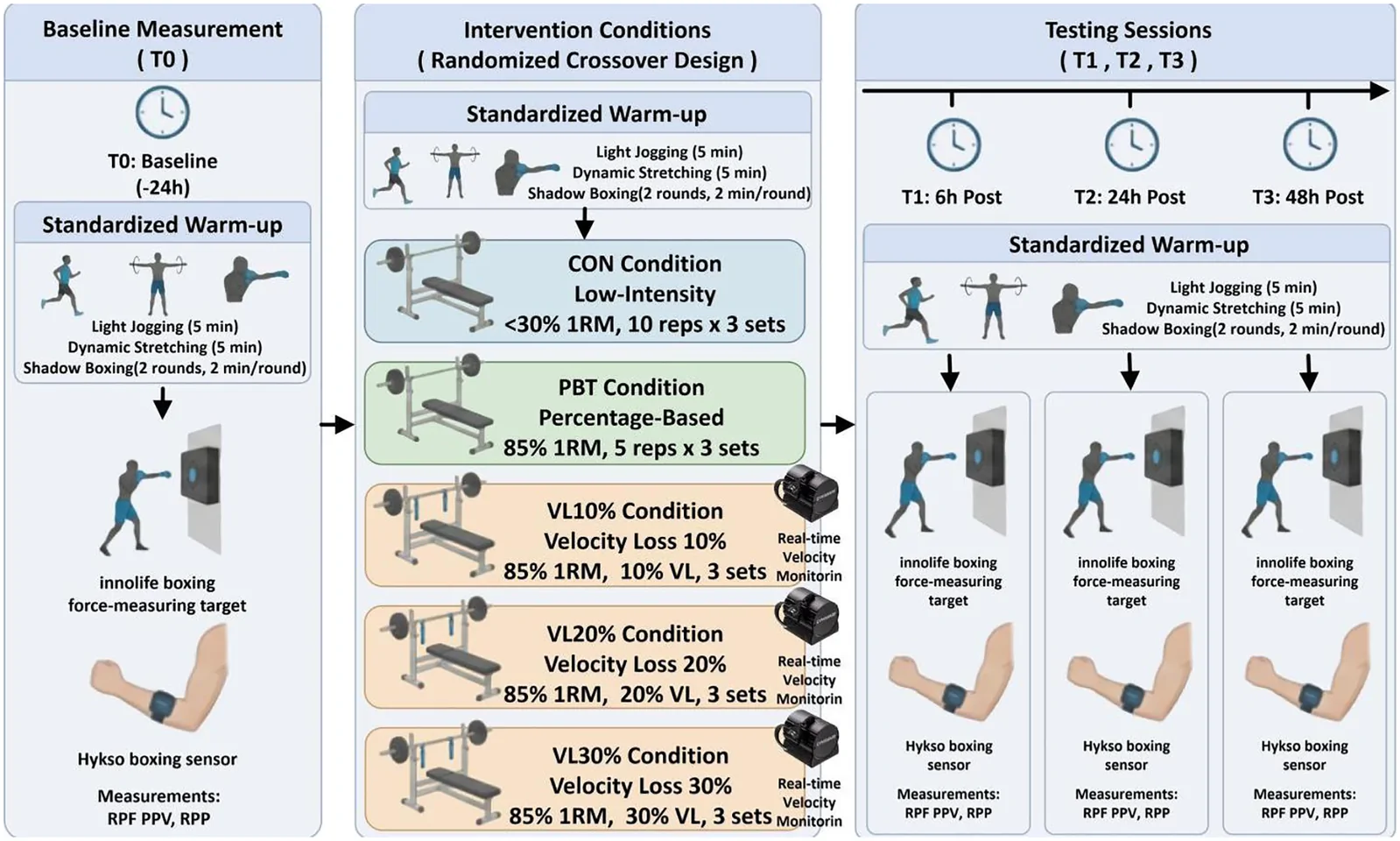
Experimental design of the randomized crossover protocol. Overview of the priming interventions (CON, PBT, VL10%, VL20%, and VL30%) and the timing of lead straight punch-performance assessments (T0, T1, T2, and T3). CON was a low-intensity active control condition consisting of bench press exercise at <30% 1RM for 3 sets of 10 repetitions. Bench press priming was performed at 85% 1RM in the PBT and VBT conditions, with VBT sets terminated at predefined velocity-loss thresholds. The primary punch-performance outcomes were relative peak punch force (RPF; N/kg), peak punch velocity (PPV; m/s), and estimated relative punch-power index (RPP; W/kg).

This study employed a randomized, balanced crossover experimental design in which each participant completed all intervention conditions in a counterbalanced order. Specifically, each participant underwent five different intervention conditions in a random order, which was determined by the 5 × 5 sequence generated by Williams’ balanced Latin square (Beattie et al., 2014; Palmer et al., 2015): (a) low-intensity active control condition (CON; 3 sets of 10 bench press repetitions at <30% 1RM); (b) percentage-based training (PBT; 3 sets of 5 repetitions at 85% 1RM); and three VBT conditions performed at 85% 1RM with sets terminated upon reaching velocity loss thresholds of (c) 10% (VL10%), (d) 20% (VL20%), or (e) 30% (VL30%) (Jukic et al., 2023). In each condition, lead straight punch performance was assessed at four standardized time points: baseline measurements (T0) were obtained 24 h prior to the intervention to establish a stable reference value under recovered conditions. Follow-up assessments were conducted at 6 h (T1), 24 h (T2), and 48 h (T3) post-intervention to reflect the commonly reported DPE window of 6–48 h. The 6 h time point was selected to examine early recovery effects, the 24 h assessment to evaluate peak delayed potentiation responses, and the 48 h measurement to determine whether performance enhancements persisted or returned toward baseline (Tsoukos et al., 2018; Niu et al., 2025). A washout period of 5 days was implemented between each condition to ensure complete recovery and to mitigate interference from residual training effects. Each condition was conducted in the morning (between 9:00 and 11:00 a.m.) to control for circadian effects. Throughout the experimental period, participants maintained their normal boxing training routine but avoided any additional high-intensity upper-body strength training for at least 24 h prior to each test day. They were also instructed to abstain from caffeine and alcohol to prevent these factors from affecting performance or recovery in the study outcomes. All tests were conducted in the same indoor facility under controlled environmental conditions (room temperature 22–24 °C, relative humidity 40–60%) to further ensure consistency and reliability of the results. To minimize potential bias, outcome assessors and data analysts were blinded to intervention condition coding during performance assessment and statistical analysis. Complete participant blinding was not possible because the interventions differed in load, repetition number, and set duration. Furthermore, intervention staff could not be blinded because they were required to monitor repetition velocity and terminate sets according to the prespecified VL thresholds. Given the crossover design, participants were instructed to replicate their 24-h dietary intake prior to each experimental condition. A standardized pre-testing meal timing was maintained across all sessions. This procedure was implemented to minimize variability in substrate availability and recovery kinetics between conditions. **Figure 1** illustrates the experimental design and flow of participants through the study.

### 2.2 Participants

An *a priori* sample-size calculation was performed using G*Power version 3.1.9.7 (Heinrich-Heine-Universität Düsseldorf, Düsseldorf, Germany). Because G*Power does not directly implement the planned five-condition crossover linear mixed-effects model, a repeated-measures ANOVA with within-subject factors was used as a pragmatic approximation of the repeated-measures structure. The expected effect size was informed by the magnitude of delayed performance enhancement reported for countermovement jump performance at 6 h and 24 h following high-intensity resistance priming (González-García et al., 2021; Niu et al., 2025). Accordingly, a moderate effect size of Cohen’s f = 0.25 was adopted. The calculation used the following parameters: F tests as the test family; repeated-measures ANOVA within factors as the statistical test; an *a priori* power analysis; α = 0.05; statistical power (1 − β) = 0.80; one group; four repeated measurements corresponding to baseline and 6 h, 24 h, and 48 h post-intervention assessments; a correlation of 0.70 among repeated measurements; and a nonsphericity correction of ϵ = 1.00. These assumptions yielded a minimum required sample size of 15 participants. To account for an anticipated attrition rate of approximately 15%, the recruitment target was increased to 18 participants. All 18 recruited participants completed the study and were included in the final sample. The sample comprised highly trained male boxers from the Shandong Province Men’s Boxing Team. Participants were aged 16–19 years (17.6 ± 0.7 years), including seven participants younger than 18 years. They had 3.2 ± 1.7 years of systematic boxing training experience and completed 27.8 ± 5.2 h of boxing training per week. The inclusion criteria were: i) According to the athlete classification framework proposed by McKay et al (McKay et al., 2022), participants were categorized as Tier 3 (highly trained/national level) athletes based on their competitive ranking and training history; ii) no significant injuries in the past three months; iii) bench press 1RM greater than body weight (Folhes et al., 2022); iv) proficiency in the lead straight punch technique (Folhes et al., 2022). The exclusion criteria included: i) presence of any cardiovascular or neuromuscular disease; ii) use of medications affecting neuromuscular function in the past three months; iii) history of upper-limb surgery or major injury within the past six months. The descriptive characteristics of the participants are presented in **Table 1**. This study received approval from the Ethics Committee of Beijing Sport University (approval number: 2025416H). Participants aged 18 years or older provided written informed consent before participation. Participants younger than 18 years provided written assent, and written informed consent was obtained from a parent or legal guardian before enrollment.

**Table 1:** Participant characteristics (mean ± SD).

Variables	Mean ± SD
Age (years)	17.6 ± 0.7
Height (cm)	179.5 ± 5.2
Body mass (kg)	67.2 ± 7.1
1RM (kg)	85.6 ± 9.8
1RM/body mass	1.3 ± 0.3
Boxing experience (years)	3.2 ± 1.7
Boxing training volume per week (hours)	27.8 ± 5.2

### 2.3 Procedures

#### 2.3.1 Familiarization sessions

One week prior to the commencement of the formal experiment, all participants completed a comprehensive familiarization session. This session included determination of bench press 1RM, technical standardization of lifting mechanics, adaptation to the velocity monitoring device, and repeated practice of the lead straight punch testing protocol. Participants were instructed and supervised to ensure consistent bench press technique (e.g., grip width, bar path, tempo control) and standardized punching mechanics during force and velocity assessments. These procedures were implemented to reduce measurement variability, ensure technical consistency across testing sessions, and minimize potential learning effects that could confound subsequent performance outcomes.

#### 2.3.2 Experimental conditions

Across all bench press conditions, the technical execution was standardized. Participants used a medium grip width and performed each repetition with a controlled eccentric phase (approximately 2 s), without pausing at the bottom position. Concentric movement was performed with maximal intended velocity, with no pause at lockout (tempo: 2/0/X/0). All sessions were supervised to ensure consistent technique. For the PBT and all VBT conditions, the prescribed load was set at 85% of each participant’s 1RM and rounded to the nearest 2.5 kg. Condition-specific volume-load was calculated at the participant level as the rounded external load multiplied by the total number of repetitions completed across the three sets. The CON condition consisted of a low-intensity active bench press protocol designed to approximate the duration of the experimental conditions while minimizing fatigue. Participants performed 3 sets of 10 repetitions at <30% 1RM, with 3 min of rest between sets (Nakamura et al., 2019). The PBT condition consisted of traditional percentage-intensity bench press strength training using a load of 85% of 1RM, completing 3 sets of 5 repetitions, with a 3-min rest interval between sets (Beaven et al., 2008). The VL10% condition consisted of bench press training at a load of 85% of 1RM, monitoring the mean concentric velocity (MCV) of each repetition in real-time using a linear position transducer. Participants completed three sets; however, no predetermined number of repetitions was prescribed. Instead, each set was terminated once the MCV of a repetition decreased by 10% relative to the fastest repetition recorded within that set. A 3-min rest interval was provided between sets. The VL20% condition followed the same load (85% 1RM), tempo, set structure (three sets), and rest interval (3 min) as the VL10% protocol, differing only in the velocity loss threshold. In this condition, each set was terminated when the MCV declined by 20% from the fastest repetition recorded within that set. Similarly, the VL30% condition employed identical loading, tempo, and rest parameters, with set termination occurring at a 30% reduction in MCV (Rodiles-Guerrero et al., 2020; Hernández-Belmonte and Pallarés, 2022; Li et al., 2024). During all VBT conditions, a linear position transducer (GymAware RS; Kinetic Performance Technologies, Canberra, Australia) was utilized to continuously monitor the MCV of each repetition in real-time. To accurately quantify intra-set velocity loss, the repetition yielding the highest MCV within a given set was established as the baseline reference. The MCV of all subsequent repetitions was instantaneously compared against this intra-set peak, and the set was immediately terminated once the velocity dropped by the prescribed threshold.

### 2.4 Testing and measurements

Lead straight punch performance was evaluated using a standardized testing protocol conducted at four time points (T0, T1, T2, and T3), as previously described. At each assessment session, following a standardized warm-up (5 min light jogging, 5 min dynamic upper-body mobility exercises, and two 2-min rounds of shadow boxing), participants performed three maximal-effort lead straight punches under controlled laboratory conditions. All punches were executed from a standardized boxing stance, with the striking target positioned at shoulder height and the starting distance individualized according to each athlete’s functional arm span and marked on the floor to ensure consistency across sessions. Adequate rest was provided between repetitions to minimize fatigue. The highest valid value from the three attempts was retained for statistical analysis. Prior to formal data collection, participants completed a same-day familiarization and verification procedure to reinforce technical consistency with the punching protocol. All assessments were conducted by the same research team, at the same facility, and using identical equipment configurations throughout the study. RPF, PPV, and RPP were designated as the primary performance outcomes. Peak punch force (PPF; N) and peak punch velocity (PPV; m/s) were used to derive RPF and RPP as described below. Detailed descriptions of signal acquisition and variable calculations are provided below.

#### 2.4.1 Relative peak punch force

Force signals during the lead straight punching test were collected using an Innolife boxing force-measuring target (InnoLife Technology, USA). The force-measuring target was positioned at shoulder height and oriented vertically to the ground, with a sampling frequency of 1000 Hz. PPF was defined as the maximum instantaneous value of the force–time curve and was expressed in newtons. RPF was calculated by dividing PPF by body mass: RPF (N/kg) = PPF (N)/body mass (kg).

#### 2.4.2 Peak punch velocity

Velocity signals were obtained using a Hykso wearable boxing sensor (Hykso Inc., USA), which has previously been validated for quantifying punch velocity and punch count in boxing research (Omcirk et al., 2021, Omcirk et al., 2023). The sensor was secured to the distal forearm, proximal to the wrist joint, using an adjustable strap to ensure stable positioning and measurement consistency across sessions. The device generated a velocity–time signal, from which PPV was defined as the maximum instantaneous velocity recorded during the punch and was expressed in meters per second (m/s).

#### 2.4.3 Estimated relative punch-power index

For each valid punch attempt, peak punch force (PPF; N) obtained from the force-measuring target and peak punch velocity (PPV; m/s) obtained from the wearable sensor were paired within the same punch attempt. RPP was calculated separately for each attempt as follows: RPP (W/kg) = [PPF (N) × PPV (m/s)]/body mass (kg).

Although PPF and PPV were obtained from the same punch attempt, force and velocity were recorded independently using separate devices and were not synchronized at the waveform level. Because peak force and peak velocity may have occurred at different instants during the punching action, RPP does not represent directly measured instantaneous mechanical power. Accordingly, RPP was interpreted as an estimated same-attempt force–velocity product, or punch-power index. The highest valid RPP value among the three punch attempts was retained for statistical analysis.

#### 2.4.4 Standardization of boxing glove donning

To mitigate the influence of equipment variability on mechanical output, we adhered to the International Boxing Association (IBA) “Technical & Competition Rules” regarding glove weight and specifications. Elite and young male boxers use 10-ounce gloves for the Minimum Weight (46–48 kg) category to the Welterweight (67 kg) category, while 12-ounce gloves are used in the Light Middleweight (71 kg) category to Super Heavyweight (>92 kg) category. Throughout the study, the brand and size of the gloves were assigned and standardized according to the participants’ weight categories, ensuring consistency throughout the entire duration of the research.

### 2.5 Statistical analyses

All statistical analyses were performed using R version 4.3.1. Linear mixed-effects models (LMMs) were fitted using the lme4 and lmerTest packages. Sum contrasts were specified, and Type III tests of fixed effects were conducted. Fixed effects included condition (CON, PBT, VL10%, VL20%, and VL30%), time (T0, T1, T2, and T3), and the condition × time interaction. A participant-specific random intercept was included to account for repeated observations and between-participant baseline differences. Models were estimated using restricted maximum likelihood. F tests used Kenward–Roger degrees-of-freedom corrections. Because the primary inferential question concerned whether each active protocol differed from the low-intensity active control condition (CON) at each assessment time point, Dunnett-adjusted estimated marginal mean contrasts were used to compare PBT, VL10%, VL20%, and VL30% with CON at T0, T1, T2, and T3.

## 3 Results

### 3.1 Training volume characteristics

The total number of repetitions completed across the three sets differed substantially among the velocity-loss conditions. Participants completed 6.2 ± 1.6 repetitions in total in VL10%, 11.7 ± 2.1 repetitions in VL20%, and 16.1 ± 3.7 repetitions in VL30%. Set-specific repetition counts are presented in **Supplementary Table S1**. Descriptively, repetition counts declined from Set 1 to Set 3 within each VL condition, whereas higher velocity-loss thresholds permitted more repetitions within each set and across the three sets in total. The PBT condition involved a fixed total of 15 repetitions (3 × 5), whereas the CON condition involved 30 repetitions (3 × 10 at <30% 1RM).

Participant-level volume-load was 1095.8 ± 125.7 kg in PBT, 454.7 ± 123.2 kg in VL10%, 860.1 ± 190.5 kg in VL20%, and 1178.3 ± 316.2 kg in VL30%. Volume-load was not reported for CON because participant-level external loads were not retained. Set-duration data were unavailable for all conditions. Thus, the VL conditions differed not only in the prescribed velocity-loss threshold but also in repetition volume and volume-load. Consequently, the independent effects of VL threshold and training dose cannot be separated.

### 3.2 Relative peak punch force

**Figure 2:**
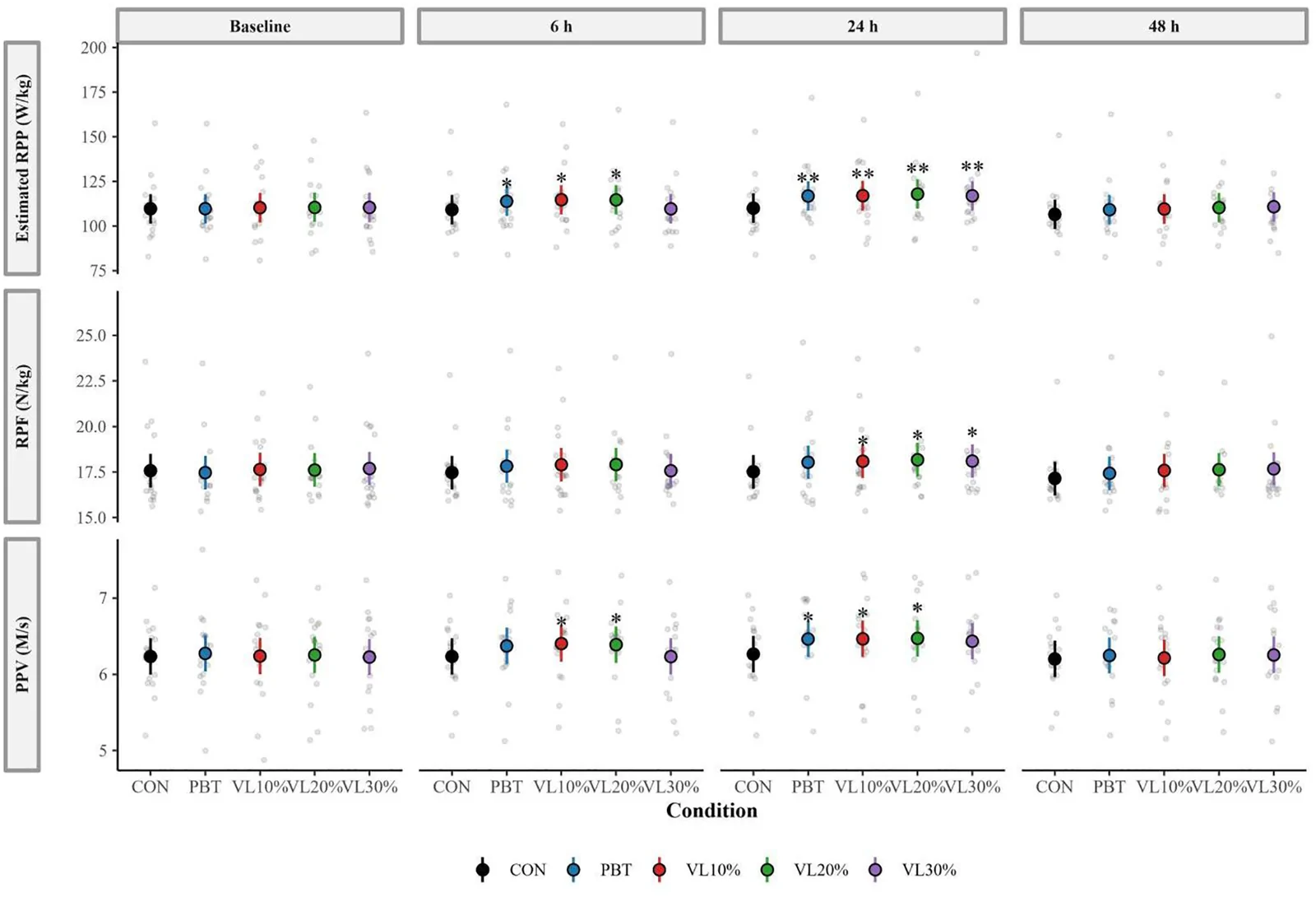
Lead straight punch performance outcomes across conditions and time points. Points and error bars represent estimated marginal means (EMMs) and 95% confidence intervals for relative peak punch force (RPF; N/kg), peak punch velocity (PPV; m/s), and estimated relative punch-power index (RPP; W/kg). Asterisks denote Dunnett-adjusted contrasts versus the low-intensity active control condition (CON) at the same time point (*p < 0.05; **p < 0.01).

A significant main effect of condition was observed (F = 4.11, p = 0.003), indicating overall differences in RPF between the intervention protocols when averaged across time points. A significant main effect of time was also detected (F = 8.83, p < 0.001), suggesting that RPF changed across the measurement occasions irrespective of condition. However, the condition × time interaction was not significant (F = 0.76, p = 0.69), indicating that the temporal pattern of change did not differ statistically between protocols. Dunnett-adjusted contrasts versus CON showed that, at 24 h post-intervention (T2), RPF values were significantly higher than CON in VL10% (MD = 0.57 N/kg, 95% CI 0.00 to 1.15; p = 0.049; g = 0.83, 95% CI 0.00 to 1.66), VL20% (MD = 0.66 N/kg, 95% CI 0.08 to 1.23; p = 0.019; g = 0.95, 95% CI 0.12 to 1.78), and VL30% (MD = 0.59 N/kg, 95% CI 0.01 to 1.16; p = 0.044; g = 0.85, 95% CI 0.02 to 1.68), whereas the PBT condition did not differ significantly from CON (p = 0.087). No significant differences between experimental conditions and CON were observed at T0, T1, or T3 (all p > 0.05). At T3, neither VL20% (p = 0.095) nor VL30% (p = 0.059) differed significantly from CON (**Figure 2**).

### 3.3 Peak punch velocity

A significant main effect of condition was observed (F = 3.79, p = 0.005). A significant main effect of time was also detected for PPV (F = 16.07, p < 0.001). However, the condition × time interaction was not significant (F = 1.07, p = 0.38). Dunnett-adjusted contrasts versus CON showed no significant differences between any intervention condition and CON at baseline (T0) or 48 h post-intervention (T3) (all p > 0.05). At 6 h post-intervention (T1), PPV was significantly greater than CON in VL10% (MD = 0.17 m/s, 95% CI 0.03 to 0.31; p = 0.015; g = 0.98, 95% CI 0.15 to 1.81) and VL20% (MD = 0.15 m/s, 95% CI 0.01 to 0.30; p = 0.030; g = 0.90, 95% CI 0.07 to 1.73), whereas the PBT condition did not reach statistical significance (p = 0.063). At 24 h (T2), PPV values were significantly higher than CON in PBT (MD = 0.20 m/s, 95% CI 0.01 to 0.38; p = 0.034; g = 0.88, 95% CI 0.05 to 1.71), VL10% (MD = 0.20 m/s, 95% CI 0.01 to 0.38; p = 0.032; g = 0.89, 95% CI 0.06 to 1.72), and VL20% (MD = 0.21 m/s, 95% CI 0.02 to 0.39; p = 0.025; g = 0.92, 95% CI 0.09 to 1.75), while the VL30% condition did not differ significantly from CON (p = 0.088; **Figure 2**).

### 3.4 Estimated relative punch-power index

A significant main effect of condition was observed (F = 6.22, p < 0.001), indicating overall differences in RPP between intervention protocols when averaged across time points. A significant main effect of time was also detected (F = 20.75, p < 0.001), demonstrating that RPP changed across the measurement occasions irrespective of condition. However, the condition × time interaction was not significant (F = 1.41, p = 0.16), suggesting that the temporal pattern of RPP responses did not differ statistically between protocols. Dunnett-adjusted contrasts versus CON showed no significant differences between any intervention condition and CON at baseline (T0) or 48 h post-intervention (T3) (all p > 0.05). At 6 h post-intervention (T1), RPP was significantly greater than CON in PBT (MD = 4.66 W/kg, 95% CI 0.17 to 9.15; p = 0.040; g = 0.86, 95% CI 0.03 to 1.69), VL10% (MD = 5.56 W/kg, 95% CI 1.07 to 10.05; p = 0.010; g = 1.03, 95% CI 0.20 to 1.86), and VL20% (MD = 5.49 W/kg, 95% CI 1.00 to 9.98; p = 0.011; g = 1.02, 95% CI 0.18 to 1.85), whereas the VL30% condition did not differ from CON (p = 0.984). At 24 h (T2), all experimental conditions demonstrated significantly higher RPP values compared with CON: PBT (MD = 6.82 W/kg, 95% CI 1.48 to 12.16; p = 0.008; g = 1.06, 95% CI 0.23 to 1.89), VL10% (MD = 7.01 W/kg, 95% CI 1.67 to 12.35; p = 0.006; g = 1.09, 95% CI 0.26 to 1.92), VL20% (MD = 7.95 W/kg, 95% CI 2.61 to 13.30; p = 0.001; g = 1.24, 95% CI 0.41 to 2.07), and VL30% (MD = 6.97 W/kg, 95% CI 1.62 to 12.31; p = 0.006; g = 1.08, 95% CI 0.25 to 1.91) (**Figure 2**). Descriptive values (mean ± SD) for each condition at each assessment time point are provided in **Supplementary Tables S2**–**S4**.

## 4 Discussion

The primary objective of this study was to examine lead straight punch performance following three VBT protocols employing different velocity-loss thresholds (VL10%, VL20%, and VL30%) and traditional PBT in highly trained boxers. Dunnett-adjusted contrasts showed that, at 6 h, VL10% and VL20% were higher than CON for PPV and RPP, whereas PBT was higher than CON for RPP. At 24 h, all active conditions were higher than CON for RPP; PBT, VL10%, and VL20% were higher than CON for PPV; and VL10%, VL20%, and VL30% were higher than CON for RPF. No active condition differed significantly from CON at 48 h. However, the condition × time interactions were not significant for RPF, PPV, or RPP, and contrasts comparing the change from baseline in each active condition with the corresponding change in CON were not performed. Accordingly, these findings indicate differences from the low-intensity active control condition at specific post-intervention time points rather than confirmed differences in change from baseline or temporal response. Moreover, because the contrasts did not directly compare the active protocols, the findings do not establish that any active protocol was superior to another.

An important consideration is that the VBT conditions differed not only in the prescribed VL threshold but also in the resulting training dose. Total repetitions increased from 6.2 ± 1.6 in VL10% to 11.7 ± 2.1 in VL20% and 16.1 ± 3.7 in VL30%. Volume-load likewise increased from 454.7 ± 123.2 kg in VL10% to 860.1 ± 190.5 kg in VL20% and 1178.3 ± 316.2 kg in VL30%. The set-specific data also showed a descriptive decline in repetitions from Set 1 to Set 3 within each VL condition (**Supplementary Table S1**). These differences in repetition volume and volume-load may have altered fatigue exposure and subsequent performance responses. Although participant-level volume-load could be reconstructed for PBT and the three VL conditions, it could not be reported for CON because participant-level external loads were not retained. Set-duration data were also unavailable. Consequently, the time-specific differences from CON observed following the VL conditions cannot be attributed to the prescribed VL threshold alone and may reflect the combined influence of VL threshold, repetition volume, volume-load, and associated fatigue exposure. Because the protocols were not volume-matched, the present study cannot isolate the independent effects of VL threshold from those of total training dose. Future studies should use volume-matched protocols and prospectively retain external-load, repetition, set-duration, and direct fatigue data.

The time-specific differences from CON observed in the current study may be compatible with several neuromuscular and metabolic mechanisms proposed in the previous literature; however, these mechanisms should be regarded as hypotheses rather than direct explanations of the present findings (Harrison et al., 2019; González-García et al., 2021; Škarabot et al., 2021). More broadly, potential resistance-training responses may involve interactions across supraspinal and spinal pathways, spinal motoneurons, motor-unit behavior, and muscular contractile and metabolic processes, rather than resulting from a single isolated mechanism (Lecce et al., 2026). Potential neural contributors discussed in previous research include changes in motor-unit recruitment and firing behavior, as well as neural drive to the working musculature (Elgueta-Cancino et al., 2022; Lecce et al., 2026). Recent evidence further highlights the importance of maximal intended movement velocity for power- and rapid-force-related adaptations. In a 4-week volume-load-matched study involving elite athletes, maximal-intended-velocity resistance training produced improvements in mean propulsive velocity across different loads and a larger increase in mean propulsive power than controlled-velocity training, despite comparable improvements in maximal strength (Lecce et al., 2025a). A subsequent study similarly showed that maximal-intended-velocity training produced favorable changes in maximal propulsive power, rate of force development, and impulse, with improvements in maximal propulsive power being positively associated with improvements in rate of force development (Lecce et al., 2025b). Although these studies examined adaptations following several weeks of resistance training rather than acute DPE, they provide relevant mechanistic context but do not demonstrate that the same processes mediated the responses observed in the present study.

Metabolic recovery, including the restoration of intramuscular energy substrates, has also been proposed as a possible contributor to subsequent high-intensity performance (Impey et al., 2016; Panteli et al., 2024). Participants were instructed to replicate their 24-h dietary intake and maintain consistent pre-testing meal timing across conditions. These procedures may have reduced between-session nutritional variability; however, they do not establish that muscle glycogen, phosphocreatine availability, or other metabolic conditions were equivalent across sessions. Nevertheless, neural drive, motor-unit behavior, muscle glycogen, phosphocreatine resynthesis, and other neuromuscular or metabolic responses were not directly measured. Therefore, the present findings cannot determine which, if any, of these mechanisms contributed to the observed time-specific performance differences. The higher punch-velocity and estimated RPP values observed relative to CON at selected 6-h and 24-h assessments following VL10% and VL20% should consequently be interpreted as time-specific performance differences rather than evidence of a specific physiological mechanism. One possible interpretation is that VL regulation may help preserve the intended high-velocity neuromuscular stimulus while managing fatigue, although this hypothesis requires confirmation using direct physiological and neuromuscular measurements.

Relative to the low-intensity active control condition, VL10% and VL20% showed higher punch-velocity and/or estimated RPP values than CON at selected 6-h and 24-h assessments. However, because direct contrasts among the active interventions were not performed, the present findings do not demonstrate that VL10% or VL20% was superior to PBT or VL30%. One possible interpretation, informed by previous research, is that velocity-loss regulation may help preserve movement velocity and concentric power output while limiting excessive fatigue accumulation (Sánchez-Medina and González-Badillo, 2011; Hernández-Belmonte and Pallarés, 2022; Cui et al., 2024). Previous studies have suggested that lower velocity-loss thresholds may provide a favorable balance between maintaining high-velocity contractions and controlling fatigue, whereas higher thresholds are generally associated with greater metabolic and neuromuscular fatigue (Pareja-Blanco et al., 2017; Rodríguez-Rosell et al., 2020; Weakley et al., 2024). Nevertheless, these mechanisms were not directly assessed in the present study. Furthermore, the VL protocols differed substantially in completed repetitions, volume-load, and accumulated training dose. Consequently, the observed pattern of time-specific differences from CON may reflect the combined influence of the prescribed VL threshold and total training dose rather than the independent effect of VL threshold alone. The present findings are therefore compatible with, but do not confirm, the hypothesis that lower VL thresholds may preserve a high-velocity training stimulus while limiting fatigue.

At 24 h post-training (T2), all active conditions were significantly higher than CON for RPP; PBT, VL10%, and VL20% were higher than CON for PPV; and VL10%, VL20%, and VL30% were higher than CON for RPF. However, these were separate comparisons with CON and do not establish superiority or inferiority among the active protocols. Within the comparisons against CON, VL30% was associated with higher RPF and RPP at 24 h. No significant differences from CON were observed for the other outcomes or time points. This pattern should not be interpreted as evidence that VL30% was statistically less effective than PBT, VL10%, or VL20%. Previous research has nevertheless reported that higher VL thresholds are often accompanied by greater training volume, fatigue accumulation, and longer recovery requirements. For example, Pareja-Blanco et al. compared 20% and 40% VL thresholds during back-squat VBT and found that the higher VL condition produced greater training volume and fatigue-related responses without superior performance outcomes (Pareja-Blanco et al., 2017). Similarly, higher VL thresholds have been associated with greater neuromuscular and metabolic stress and slower recovery than lower thresholds (Rodríguez-Rosell et al., 2021; Hernández-Belmonte and Pallarés, 2022; Jukic et al., 2023). This interpretation is further supported by Weakley et al., who, in a crossover design comparing 10%, 20%, and 30% VL thresholds, demonstrated that the 30% condition elicited a significantly greater acute fatigue response that persisted for up to 24 h relative to the lower VL protocols (Weakley et al., 2024). However, these studies provide contextual support rather than a direct explanation of the present results. Because the VL conditions in the current study differed substantially in completed repetitions and accumulated training dose, the pattern of time-specific differences from CON observed following VL30% may reflect the combined effects of VL threshold, training volume, and fatigue exposure. Collectively, the present findings and previous literature highlight the importance of carefully regulating both velocity loss and total training dose during pre-competition resistance priming, while further volume-matched studies with direct comparisons among active protocols are needed to determine whether specific VL thresholds produce meaningfully different delayed performance responses.

It is widely acknowledged that the lead straight punch in boxing is not solely dependent on upper-limb strength and velocity, but rather involves a coordinated sequence of lower-body explosive power, core stability, and whole-body kinematics (Dinu and Louis, 2020; Liu et al., 2022). The power for punching is generated from the lower limbs and transmitted sequentially through the kinetic chain, utilizing core stability for effective force transfer to the upper extremities, ultimately culminating in impactful striking performance (Dunn et al., 2022). The present study was limited to the bench press as an upper-body stimulus. Future research could extend the VBT framework to lower-body or whole-body exercises (e.g., squats, power cleans) to simultaneously enhance the power output across the entire kinetic chain (Beattie and Ruddock, 2022; Yi et al., 2022).

Despite its practical implications for load regulation in boxing-specific strength preparation, the present study has several limitations. First, the sample comprised a homogeneous cohort of highly trained male boxers, without the inclusion of female athletes or stratification by weight category. Given the potential influence of sex- and mass-related differences in neuromuscular characteristics, future investigations should examine more diverse populations to enhance the external validity and generalizability of the findings. In addition, seven participants were younger than 18 years, and individual differences in biological maturation may have contributed to variability in fatigue, recovery, and punch-performance responses. Second, the resistance-priming stimulus was limited to the bench press, which, although relevant to upper-body force production, does not fully represent the integrated contribution of the lower limbs and trunk within the kinetic chain during punching. Incorporating velocity-based exercises targeting the lower body and whole-body power, such as squats, deadlifts, or ballistic movements, may provide a more comprehensive understanding of how priming strategies influence boxing performance. Moreover, although lead straight punch metrics provide a controlled and sport-specific proxy for boxing performance, they represent only one component of competitive success. Future research should incorporate more ecologically valid assessments, including combination punching, reactive tasks, sparring simulations, and competition-based performance indices. Third, the VL conditions were not matched for training dose. Higher VL thresholds resulted in greater repetition counts within each set, greater total repetition volume, and greater volume-load, likely increasing fatigue exposure. Participant-level volume-load could be reconstructed for PBT and the three VL conditions but not for CON because the actual participant-level external loads used in CON were not retained. Set-duration data were unavailable for all conditions. Therefore, the effects of the prescribed VL threshold cannot be separated from those of total training dose and associated fatigue exposure. The time-specific findings observed following VL30% may consequently reflect the combined influence of the higher VL threshold, greater repetition volume, and greater volume-load. Future studies should compare VL thresholds under volume-matched conditions and prospectively retain external-load, repetition, set-duration, and direct fatigue data. Fourth, no direct neuromuscular or metabolic measurements were collected. Therefore, the proposed roles of neural drive, motor-unit recruitment, glycogen restoration, phosphocreatine resynthesis, and related processes should be regarded as literature-based hypotheses rather than mechanisms demonstrated by the present data. Fifth, the estimated relative punch-power index (RPP) was calculated from peak punch force and peak punch velocity values obtained from the same punch attempt but recorded independently using separate devices without waveform-level synchronization. Because peak force and peak velocity may have occurred at different instants during the punching action, RPP should be interpreted as an estimated same-attempt force–velocity product rather than a direct measurement of instantaneous mechanical power. Future studies should use synchronized force–velocity acquisition systems to directly quantify instantaneous punch power. Sixth, formal reliability and measurement-error indices for RPF, PPV, and RPP, including the intraclass correlation coefficient, coefficient of variation, standard error of measurement, and minimal detectable change, were not available and could not be calculated retrospectively. The trial-level data required for a retrospective reliability analysis were not retained. Although the punching protocol, testing procedures, and equipment configurations were standardized, it therefore remains uncertain whether all statistically significant differences, particularly the smaller mean differences, exceeded the normal measurement variability of the testing system. This uncertainty limits conclusions regarding the practical importance of the observed differences. Future studies should establish test–retest reliability and prespecify measurement-error or smallest-worthwhile-change thresholds for these boxing-specific outcomes. Seventh, sleep duration and sleep quality were not assessed before each experimental session. Although participants were instructed to maintain their habitual lifestyle and replicate their dietary intake before each trial, uncontrolled between-session variation in sleep may have influenced recovery status, neuromuscular readiness, and subsequent punch-performance responses.

Finally, the study examined only short-term responses following isolated priming sessions; therefore, the long-term effects of repeated velocity-regulated priming within a periodized training program remain unknown.

## 5 Conclusion

At 6 h post-intervention, RPP was higher than CON following PBT, VL10%, and VL20%, whereas PPV was higher than CON following VL10% and VL20%. At 24 h, RPP was higher than CON following all active conditions; PPV was higher than CON following PBT, VL10%, and VL20%; and RPF was higher than CON following VL10%, VL20%, and VL30%. No active condition differed significantly from CON at 48 h. However, because the condition × time interactions were not significant and change-from-baseline contrasts versus CON were not performed, these findings should be interpreted as differences from the low-intensity active control condition at specific post-intervention time points rather than evidence of greater change from baseline or a distinct temporal response. The analyses also did not directly compare the active protocols and therefore do not establish the superiority of any active protocol. In addition, unequal repetition counts and volume-loads across the VL conditions prevent the effects of VL threshold from being separated from those of training dose and fatigue exposure. Accordingly, the present study does not identify an optimal or superior VL range.

## Data Availability

The original contributions presented in the study are included in the article/**Supplementary Material**, further inquiries can be directed to the corresponding author/s.
