# Supplementary material for: Delayed lead straight punch performance following resistance priming with different velocity-loss thresholds in highly trained boxers
Source: Front Physiol. 2026 Sep 3;17:1828882. doi: 10.3389/fphys.2026.1828882 (PMC13581863; doi:10.3389/fphys.2026.1828882)
Supplement: Supplementary file 1 [file Supplementaryfile1.docx]

Supplementary Material

Supplementary Table S1. Set-specific repetitions, total repetitions, and volume-load during the bench-press priming conditions

| Condition | Relative load | Set 1 | Set 2 | Set 3 | Total across three sets | Volume-load (kg) |
| --- | --- | --- | --- | --- | --- | --- |
| CON | <30% 1RM | 10 | 10 | 10 | 30 | Not reported |
| PBT | 85% 1RM | 5 | 5 | 5 | 15 | 1095.8 ± 125.7 |
| VL10% | 85% 1RM | 2.3 ± 0.7 | 2.1 ± 0.8 | 1.8 ± 0.6 | 6.2 ± 1.6 | 454.7 ± 123.2 |
| VL20% | 85% 1RM | 4.3 ± 0.8 | 3.9 ± 1.1 | 3.5 ± 1.3 | 11.7 ± 2.1 | 860.1 ± 190.5 |
| VL30% | 85% 1RM | 6.1 ± 1.6 | 5.3 ± 1.5 | 4.7 ± 1.7 | 16.1 ± 3.7 | 1178.3 ± 316.2 |

Note. Repetition counts for CON and PBT were fixed by the experimental protocol, whereas repetition counts for the VL conditions and all reported volume-load values are presented as mean ± SD. For each participant, total repetitions in the VL conditions were calculated by summing the repetitions completed across the three sets. Volume-load was calculated at the participant level as the external load multiplied by the total number of repetitions completed across the three sets. Loads in PBT and the VL conditions corresponded to 85% 1RM rounded to the nearest 2.5 kg. Volume-load for CON was not reported because participant-level external loads were not retained. Set-duration data were unavailable. CON, low-intensity active control; PBT, percentage-based training; VL, velocity loss; 1RM, one-repetition maximum.

Supplementary Table S2. Descriptive values of relative peak punch force (RPF; N/kg) following the control, percentage-based training, and velocity-based training protocols at baseline and post-intervention time points (mean ± SD).

| Time point | CON | PBT | VL10% | VL20% | VL30% |
| --- | --- | --- | --- | --- | --- |
| T0 | 17.58 ± 2.03 | 17.47 ± 1.83 | 17.64 ± 1.66 | 17.61 ± 1.55 | 17.69 ± 2.19 |
| T1 | 17.47 ± 1.64 | 17.82 ± 2.04 | 17.90 ± 2.00 | 17.91 ± 1.84 | 17.57 ± 1.85 |
| T2 | 17.52 ± 1.60 | 18.04 ± 2.21 | 18.09 ± 2.04* | 18.17 ± 1.82* | 18.10 ± 2.37* |
| T3 | 17.15 ± 1.46 | 17.43 ± 1.83 | 17.59 ± 1.94 | 17.63 ± 1.37 | 17.67 ± 2.09 |

Note. Values are presented as mean ± SD. * Significantly different from CON at the same time point based on Dunnett-adjusted contrasts (p < 0.05).

Supplementary Table S3. Descriptive values of peak punch velocity (PPV; m/s) following the control, percentage-based training, and velocity-based training protocols at baseline and post-intervention time points (mean ± SD).

| Time point | CON | PBT | VL10% | VL20% | VL30% |
| --- | --- | --- | --- | --- | --- |
| T0 | 6.25 ± 0.47 | 6.27 ± 0.53 | 6.24 ± 0.58 | 6.25 ± 0.52 | 6.23 ± 0.53 |
| T1 | 6.23 ± 0.43 | 6.37 ± 0.49 | 6.40 ± 0.48* | 6.39 ± 0.48* | 6.23 ± 0.46 |
| T2 | 6.26 ± 0.46 | 6.46 ± 0.44* | 6.46 ± 0.53* | 6.47 ± 0.54* | 6.43 ± 0.49 |
| T3 | 6.20 ± 0.51 | 6.25 ± 0.45 | 6.21 ± 0.49 | 6.26 ± 0.47 | 6.25 ± 0.53 |

Note. Values are presented as mean ± SD. * Significantly different from CON at the same time point based on Dunnett-adjusted contrasts (p < 0.05).

Supplementary Table S4. Descriptive values of the estimated relative punch-power index (RPP; W/kg) following the control, percentage-based training, and velocity-based training protocols at baseline and post-intervention time points (mean ± SD).

| Time point | CON | PBT | VL10% | VL20% | VL30% |
| --- | --- | --- | --- | --- | --- |
| T0 | 109.72 ± 16.16 | 109.72 ± 16.00 | 110.30 ± 16.58 | 110.41 ± 15.85 | 110.40 ± 18.80 |
| T1 | 109.16 ± 14.83 | 113.81 ± 18.15* | 114.71 ± 16.55* | 114.65 ± 16.97* | 109.64 ± 15.96 |
| T2 | 109.97 ± 15.08 | 116.79 ± 18.46* | 116.98 ± 16.78* | 117.93 ± 18.58* | 116.94 ± 22.29* |
| T3 | 106.55 ± 13.61 | 109.15 ± 16.54 | 109.52 ± 16.73 | 110.30 ± 11.34 | 110.79 ± 18.84 |

Note. Values are presented as mean ± SD. * Significantly different from CON at the same time point based on Dunnett-adjusted contrasts (p < 0.05).
